# Miscellany

**Published:** 1888-01

**Authors:** 


### Miscellany.

Antiseptic Treatment of Intestinal Affections.—In an article on "Intestinal Antiseptics," by D. N. Kinsman, M. D., appearing in the *Journal of the American Medical Association*, July 3, 1886, the author points out that the natural processes of fermentation and putrefaction going on in normal digestion are so changed in dyspepsia and other forms of intestinal diseases as to produce poisonous alkaloids, which are the cause of the symptoms developed in such disorders.

The researches of Prof. Vaughn, of the University of Michigan, in which tyrotoxicon has been shown to be the cause of ice-cream poisoning, which are still fresh in the minds of medical readers, have thrown still more light on the etiology of intestinal affections, and made apparent the importance of intestinal antiseptics as a method of treatment.

To facilitate such treatment, we learn that Parke, Davis & Co. have recently added to their list an intestinal antiseptic pill, the formula of which is as follows: Mercury protiodide, 1/8 gr.; podophyllin, 1-16 gr.; aloin, 1-16 gr.; ext. nux vomica, 1-16 gr.; ext. henbane, 1-16 gr.

---

That Terrible Disease—The Divers.—Governor Taylor, of Tennessee, recently heard of a colored minister who preached a sermon on the text: "And the multitudes came to Him and He healed them of divers diseases.'' Said he: "My dying congregation, this is a terrible text! Disease is in the world! The small-pox slays its hundreds, the cholera its thousands, and the yellow fever its tens of thousands, but, in the language of the text, if you take the divers, you are gone. These earthly doctors can cure the small-pox, cholera and yellow fever, if they get there in time, but nobody but the good Lord can cure the divers.''

---

Ninety Tape–Worms and One Girl.—Dr. Roux, surgeon of the Cantonal Hospital, in Lausaune, reports a case in which the patient, a girl aged twenty-two and a-half years, discharged (after two six-gramme doses of extract of male fern,) at least ninety bothriocephali lati. The worms passed out in a bundle, the patient assisting the delivery by tearing the package with both hands, at the same time uttering shrieks like a woman in labor. Except some slight phenomena (such as occasional headaches, vivid dreams, semi-somnambulism), the patient did not present any morbid symptoms.

---

The following is recommended in the *Révue de Thérapeutique* as a nutrient and sedative enema:

| | | | |
|---|---|---|---|
| ℞ | Bouillon of beef . . . . . . . . . . . . | ℥ iij. ʒj. | |
| | Eggs . . . . . . . . . . . . . . . . . . | j. | |
| | Bordeaux wine . . . . . . . . . . . | ʒ vj. gtt. xv. | |
| | Sodium bicarbonate . . . . . . . . . | gr. viij. | |
| | Tincture of opium . . . . . . . . . . | gtt. iv. | |
| | Chloride of sodium . . . . . . . . . . | gr. iij. | |
| | Peptone . . . . . . . . . . . . . . . . | ʒ iv. | M. |

---

Sick Man (gloomily)—"If I should die, dear, what in the world would become of you and the children?''

Wife (cheerfully)—"Now, you must not worry, John; the doctor says it is bad for you. I'll find some one to take care of us.'' The sick man got well.

---

It is claimed that in Cincinnati a doctor in a close carriage can persuade people that he knows much more than a doctor on foot.

An Expert.—" I suppose," said the stranger, as the group of tired travelers were discussing the nature of the malady from which the German Crown Prince is suffering. " I suppose I have had as much experience in the treatment of malignant and rapid throat troubles as any in America." "Ah, you are a physician, then?" "No," said the stranger, " not exactly, but for six years I was chairman of a Texas vigilance committee."

---

The editor of the *Pacific Medical Record* is rather severe upon the Gynæcologists when he says: It is safe to say that more than half the revenue of the physicians of the world is derived from the treatment of females; and if we say that nine-tenths of the number thus treated have no need of that treatment, and that it works actual harm, we shall utter the truth.

---

It is difficult to make the whole medical profession happy on the subject of International Congresses. A leading French medical journal considers the selection of Berlin, as the place of the next meeting, a positive insult to France, and adds that the Congress will not be an international one, but merely a German reunion.

---

A writer in the *Kansas City Medical Index* says that the fees of doctors are ridiculously low in St. Louis. A few physicians charge two dollars a visit, but most visits are made for one dollar, and many for fifty cents. Office practice is from fifty cents to one dollar. Much office work is done for twenty-five cents.

---

Gonorrhœa and Sterility.—Dr. William Goodell says: Personally, I cannot recall a case in which a woman bore a child after suffering with gonorrhœa. Strumpets rarely become pregnant, for most of them have had this affection.

---

Drunkenness and delirium tremens have so increased in Berlin, that the medical profession is considering the necessity of recommending the government to reduce the number of places where intoxicating liquors are now sold.

---

Doctors in Russia.—A recent report of the members of the profession, in proportion to the population in Russia, gave one doctor to 18,000 people. This is the only civilized country where overcrowding is not present.

